# Cereal progenitors differ in stand harvest characteristics from related wild grasses

**DOI:** 10.1111/1365-2745.12905

**Published:** 2017-11-27

**Authors:** Catherine Preece, Natalie F. Clamp, Gemma Warham, Michael Charles, Mark Rees, Glynis Jones, Colin P. Osborne

**Affiliations:** ^1^ Department of Animal and Plant Sciences University of Sheffield Sheffield UK; ^2^ CREAF Cerdanyola del Valles Spain; ^3^ Department of Archaeology University of Sheffield Sheffield UK; ^4^ Institute of Archaeology University of Oxford Oxford UK

**Keywords:** competition, domestication, fertile crescent, harvest traits, origins of agriculture, plant development and life‐history traits, seed size, yield

## Abstract

1. The domestication of crops in the Fertile Crescent began approximately 10,000 years ago indicating a change from a hunter‐gatherer lifestyle to a sedentary, agriculture‐based existence. The exploitation of wild plants changed during this transition, such that a small number of crops were domesticated from the broader range of species gathered from the wild. However, the reasons for this change are unclear.
2. Previous studies have shown unexpectedly that crop progenitors are not consistently higher yielding than related wild grass species, when growing without competition. In this study, we replicate more closely natural competition within wild stands, using two greenhouse experiments to investigate whether cereal progenitors exhibit a greater seed yield per unit area than related wild species that were not domesticated.
3. Stands of cereal progenitors do not provide a greater total seed yield per unit ground area than related wild species, but these crop progenitors do have greater reproductive efficiency than closely related wild species, with nearly twice the harvest index (the ratio of harvested seeds to total shoot dry mass).
4. These differences arise because the progenitors have greater seed yield per tiller than closely related wild species, due to larger individual seed size but no reduction in seed number per tiller. The harvest characteristics of cereal progenitors may have made them a more attractive prospect than closely related wild species for the early cultivators who first planted these species, or could suggest an ecological filtering mechanism.
5. *Synthesis*. Overall, we show that the maintenance of a high harvest index under competition, the packaging of seed in large tillers, and large seeds, consistently distinguish crop progenitors from closely related wild grass species. However, the archaeological significance of these findings remains unclear, since a number of more distantly related species, including wild oats, have an equally high or higher harvest index and yield than some of the progenitor species. Domestication of the earliest cereal crops from the pool of wild species available cannot therefore be explained solely by species differences in yield and harvest characteristics, and must also consider other plant traits.

## 1 INTRODUCTION

Wheat and barley were domesticated approximately 10,000 years ago in the Fertile Crescent region of western Asia, heralding a fundamental change in human society: the transition from subsistence, based on hunting and gathering, to an agricultural way of life. The mechanisms through which this critical transition occurred are debated (e.g. Abbo, Lev‐Yadun, & Gopher, 2014; Bar‐Yosef, 2011; Cohen, 2009; Fuller, Allaby, & Stevens, 2010; Hayden, 2009; Willcox, Nesbitt, & Bittmann, 2012). Early archaeological sites within the Fertile Crescent provide evidence of a large number of plant species, indicating that a wide variety of potentially edible plants were available at this time. However, of these, very few became domesticated crops (Savard, Nesbitt, & Jones, 2006; Weiss, Wetterstrom, Nadel, & Bar‐Yosef, 2004). Understanding why these species became domesticated, while others did not, provides useful insights into the important question of how and why agriculture originated (Price & Bar‐Yosef, 2011). Recently there has been increasing recognition that research into fundamental ecological concepts, such as the evolution of crop traits during domestication, can provide crucial insights that can help to tackle the challenge of global food security (Bardgett & Gibson, 2017; Milla, García‐Palacios, & Matesanz, 2017). Thus, a fuller understanding of the domestication process may also illuminate some of the constraints that have shaped our modern crop cultivars, and the potential to overcome these by breeding with wild relatives.

Early agriculture was founded on eight crops (einkorn and emmer wheat, barley, pea, lentil, chickpea, bitter vetch and flax), with two additional crops (oats and rye) adopted, probably at a later date (Zohary, Hopf, & Weiss, 2012). Although a few other early crop domestications (including other cereal crops) have been suggested, these are contentious (Abbo, Lev‐Yadun, Heun, & Gopher, 2013; Fuller, Willcox, & Allaby, 2012; Zohary et al., 2012), and the number of potential early domesticated species remains small compared to the range of available wild plant species. Possible reasons why particular wild species were domesticated (hereafter named “crop progenitors”), whilst others were not (hereafter named “other wild species”) include intentional selection by early farmers on the basis of traits that were deemed desirable, and unconscious selection whereby crop progenitors out‐competed other wild species in environments influenced by people, increasing the probability that they would be harvested and cultivated (Abbo et al., 2014; Cunniff et al., 2014; Fuller et al., 2012; Zohary, 2004).

Long‐held assumptions about the origins of agriculture in the Fertile Crescent tend to emphasize seed size as a defining trait for crop progenitors (Blumler, 1998), perhaps because this is a trait that is well‐preserved in the archaeobotanical record (Purugganan & Fuller, 2011). However, recent work has sought to test these assumptions. In one study that assessed plant traits within an assemblage of species collected and used as food sources in the Fertile Crescent, crop progenitors were estimated to have a higher potential seed yield than other wild species (Cunniff et al., 2014). Conversely, a study which directly studied yield using a larger number of species found that, although crop progenitors did have larger seeds than other wild species, this did not translate into a greater yield per plant, or even greater above‐ground biomass, when plants were grown individually without competition from neighbours (Preece et al., 2015). Moreover, when grown without competition, crop progenitors did not differ from other wild species across many additional traits, including allocation to reproductive tissue or timing of flowering (Preece et al., 2015). However, cereal crop progenitors did have less than half the number of spikes per plant than other wild species, leading to the proposal that cereal progenitors have a different growth form to other grasses, which may provide higher yields when these plants grow in stands. Indeed, in their wild habitats, the exploited species were likely to have been found growing naturally in stands, as in the modern Fertile Crescent (Harlan, 1967).

Characteristics that are beneficial for plants growing under competition in wild stands are not necessarily the same as for isolated plants. Thus, the value to gatherers of wild plants in stands could vary among species, which may be important for explaining why particular species were taken into cultivation. These explanations are grounded in optimal foraging theory where foragers rank food items according to their energetic value relative to harvesting and processing costs (Parker & Maynard Smith, 1990; Smith, 1983; Stephens & Krebs, 1986). However, there continues to be intense debate among researchers as to whether optimal foraging theory is relevant for understanding the development of agriculture (Gremillion, Barton, & Piperno, 2014a,b; Mohlenhoff, Coltrain, & Codding, 2015; Smith, 2014; Zeder, 2014). For people gathering seeds from the wild, the amount of grain that could be harvested from a stand might be an important determinant of which species were selected for cultivation. For example, the greater efficiency arising from a higher yield collected from a smaller area of land could lead to a greater quantity of stored grain, including seed that could be preserved for re‐planting. Despite uncertainties surrounding the factors involved in early decisions about which species to cultivate, the traits of plants growing in stands are likely to differ from those grown as individuals.

**Figure 1:**
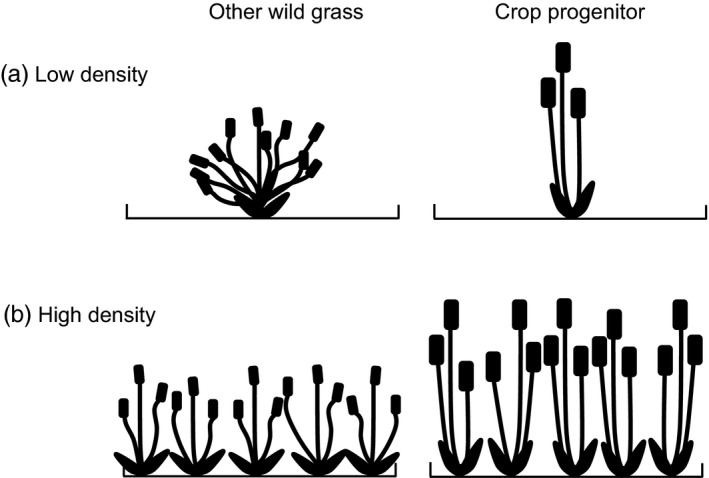
Hypothetical changes in tillering under competition for a wild grass and a cereal crop progenitor. (a) When growing at low density without competition, the crop progenitor has fewer tillers than the related wild grass. (b) At high density under competition, the related wild grass must reduce its tiller number, whereas the erect form of the crop progenitor means it can maintain its tillers

Tillering, the production of side shoots in grasses, is highly plastic under competition (Sadras & Slafer, 2012). It is important for yield in stands since, at low densities, more tillers are produced per plant to occupy space and capture light, thereby compensating for low plant densities (Evans, 1959; Sadras & Slafer, 2012). Conversely, at high densities, yield increases are halted by tiller mortality and competition for space and light (Weiner & Freckleton, 2010). Previous work on Fertile Crescent grasses has shown that cereal crop progenitors produce fewer tillers than other wild species when released from competition at low density (Preece et al., 2015). Under competition we would expect all species to experience a reduction in tiller numbers per plant, and a corresponding reduction in yield per plant. However, since crop progenitors have fewer tillers per plant, we expect this reduction to be less pronounced in general, enabling these species to potentially grow together more densely (see Figure 1).

To measure the harvest traits of Fertile Crescent grasses grown under intraspecific competition and calculate their yield per unit ground area, as experienced in wild stands, we established a greenhouse experiment with 13 species of grasses. These consisted of cereal crop progenitors and other wild species found at hunter‐gatherer sites (Preece et al., 2015). We used two ways to standardize our comparisons of total seed production (reproductive output) per ground area. First, by standardizing seed mass sown per area, and secondly by standardizing seed number sown per area. Our hypotheses were that: (1) crop progenitors would have higher seed yields under competition than other wild species; (2) tillering of crop progenitors would be reduced less by competition than that of other wild species; (3) the harvest index (the ratio of harvested grain to total shoot dry mass) of crop progenitors would be higher than that of other wild species.

## 2 MATERIALS AND METHODS

### 2.1 Plant material

#### 2.1.1 Equal seed mass experiment

In this experiment we grew 13 grass species, including wild progenitors of the major cereal crop species domesticated in the Fertile Crescent. We also included a range of other wild grass species from the same region which were never domesticated. The crop progenitors were *Hordeum vulgare* ssp. *spontaneum* (barley), *Triticum monococcum* ssp. *aegilopoides* (einkorn wheat) and *Triticum dicoccum* ssp. *dicoccoides* (emmer wheat). These three species constitute a “short list” of known crop progenitors, and for the data analyses, they were compared with the remaining ten species, termed “other wild species”.

Two of the other wild species have also been proposed as putative progenitors. Firstly, *Secale vavilovii* (progenitor of rye) was probably domesticated at a much later date. Secondly, *Triticum araraticum* may have been domesticated early (Jones, Valamoti, & Charles, 2000), although modern domesticated plants are known only from Georgia (as *Triticum timopheevii* which is no longer grown). To test whether their inclusion as crop progenitors changed our findings, we also combined these species with the three aforementioned cereal species as a progenitor “long list”, in a comparison with the remaining eight wild species.

The selection of the other wild species was made on the basis of their presence (or the presence of the genus to which they belong) in an archaeobotanical database (compiled as a result of several projects, see acknowledgements), which collates all published, and some unpublished, archaeobotanical reports for Late Epipalaeolithic and Pre‐Pottery Neolithic sites throughout the Fertile Crescent. These species were *Aegilops speltoides*,* Avena fatua*,* Avena sterilis*,* Bromus brachytsachys*,* Eremopyrum bonaepartis*,* Hordeum marinum* ssp. *gussoneanum*,* Phalaris paradoxa* and *Secale strictum*. All seeds were provided by the National Plant Germplasm System (United States Department of Agriculture) (see Supporting information Table S1). Where possible, two accessions were used per species, collected predominantly from western Asia, and chosen to span the range of seed size within each species.

#### 2.1.2 Equal sowing density experiment

We used same three crop progenitor species (short list) as in the equal seed mass experiment. Here, there were six other wild species, namely *A. speltoides*,* A. fatua*,* A. sterilis*,* H. marinum* ssp. *gussoneanum*,* H. murinum* ssp. *glaucum* and *P. paradoxa*. Between one and 14 accessions, originally collected from western Asia, were used for each species depending on availability. Seeds were obtained from various sources, including the National Plant Germplasm System (United States Department of Agriculture, Beltsville, MD, USA), the John Innes Centre Germplasm Resources Unit (Norwich, UK) and IPK Gatersleben Genebank (Stadt Seeland, Germany) (see Table S2).

### 2.2 Growth conditions

#### 2.2.1 Equal seed mass experiment

For the equal seed mass experiment, seeds were weighed individually after the outer glumes were removed. In late May 2012, they were germinated in a 1:1 mixture of John Innes no. 2 compost (LBS Garden Warehouse, Lancashire, UK) and Chelford 52 washed sand (Sibelco UK Ltd, Cheshire, UK), in a controlled‐environment growth cabinet (Conviron BDW 40, Conviron, Winnipeg, Manitoba, Canada), with conditions set to approximate the growing season in the Fertile Crescent. Temperature was 20°C/10°C (day/night), with an 8 hr photoperiod and photosynthetic photon flux density (PPFD) of 300 μmol m^−2^ s^−1^.

Following germination, when seedlings reached the two‐leaf stage, they were transferred to a second cabinet at 4°C (with the same light regime) for a 6–8 week vernalization treatment to stimulate flowering. Once vernalization was completed, plants were moved in July 2012 to a glasshouse (Arthur Willis Environment Centre, Sheffield, UK), and individuals planted in monocultures within 11 L square pots (21 cm × 21 cm × 25 cm) again with a 1:1 mixture of John Innes no. 2 compost and Chelford 52 washed sand. Each pot contained 0.4 g seed, therefore the number of seedlings per pot varied depending on the mean seed mass of the accession. As a reference, the largest‐seeded species, *S. vavilovii* had 11 or 14 individuals per pot (depending on the accession), and the smallest seeded species, *E. bonaepartis*, had 181 or 266 individuals per pot. The minimum recommended dose of Osmocote Pro slow release fertilizer was also added to each pot. Temperature was maintained at 24°C/15°C (day/night) with a 12‐hr photoperiod. The glasshouse was naturally sunlit during the high light conditions of summertime, with additional light provided on cloudy days. A subset of spikes (at least five per plant) was covered with translucent, cellophane crossing bags (Focus Packaging and Design Ltd, Scunthorpe, UK), to prevent seed dispersal prior to measurements.

#### 2.2.2 Equal sowing density experiment

For the equal sowing density experiment, which took place from November 2015 until March 2016, seeds were weighed and germinated on filter paper, then kept in a growth chamber 20°C/10°C (day/night) (Sanyo, Panasonic, Etten Leur, The Netherlands). Once germinated, the seedlings were placed on a moist 1:1 mixture of John Innes no. 3 compost and Chelford 52 washed sand, then vernalized for 6–8 weeks at 7°C. The plants were then transferred to the glasshouse and moved into 11 L square pots containing 1:1 mix of John Innes no. 2 and Chelford 52 washed sand and Osmocote fertilizer as with the first experiment.

In this experiment, 20 individuals of the same species were planted into each pot, resulting in a density of 500 plants m^−2^, ensuring that the plants experienced strong competition. The plants were then grown at 20/15°C (day/night) with a 14 hr photoperiod. During their growth, plants were watered when the soil surface became dry, usually every other day. After flowering was complete, the frequency of watering was reduced to fit with the lowered requirements of the plants. To avoid seed dispersal, the spikes of focal plants were covered in handmade muslin cloth bags. Approximately 4 weeks after replanting, aphids were seen on the plants, which were treated with a systemic pesticide (“Chess WG”, Syngenta). After this, *Chrysoperla carnea* and *Aphidius colemanii* were applied fortnightly as a biocontrol. Plants were grown until maturity in late April, except for *Hordeum spontaneum*, which were disposed of 127 days after germination due to a suspected mildew infection.

### 2.3 Experimental design and measurements

#### 2.3.1 Equal seed mass experiment

The equal seed mass experiment used a randomized block design with six blocks in total. Each block contained one pot of each species where possible. Maximum culm height was measured at maturity for the tallest plant per pot. Seeds were harvested as soon as they were ripe (prior to shattering). One replicate of *T. monococcum* ssp. *aegilopoides* produced no flowers (on any of the individuals), perhaps due to insufficient light in that part of the glasshouse, and was therefore not included in the subsequent analysis of seed yield. At maturity, after 2 months of growth, the number of fertile tillers per plant was counted for a focal plant in a subset of the replicates for each species (between one and four replicates per species). Then above‐ground biomass was harvested for all plants, and divided into vegetative and reproductive tissues. Harvested biomass was oven dried at 40°C for 3 days prior to weighing. Seeds were separated from other reproductive biomass and weighed to give total seed yield, and the total seed number per pot was also recorded. Yield per plant was calculated by dividing by the number of individuals per pot.

#### 2.3.2 Equal sowing density experiment

This experiment also used a randomized block design, with one pot of every species randomly allocated to each of six blocks, giving six replicates per species. In each pot, two focal plants were chosen by their position in the centre of the pots. Throughout the growth period, several traits were measured twice weekly for the focal plants. These included tiller number, maximum height, the proportion of fertile tillers and survival of individuals per pot. In addition to these measurements, stem diameter was recorded using calipers once flowering had occurred. Potential seed yield for individual plants was calculated by multiplying the number of seeds per spike by the average mass of the planted seed and the number of fertile tillers (in which seeds were developing) for each species. This method of estimation was based on data from Preece et al. (2017) showing a strong 1:1 correlation between the individual mass of seeds planted and harvested. By using this value and the number of plants surviving per pot, seed yield per pot (potential stand yield) could be estimated. As final biomass was not measured, HI could not be calculated in this experiment.

### 2.4 Statistical analyses

We analysed data in r (R Core Team, 2016), accounting for phylogeny. A phylogenetic tree including all of the study species was inferred using BEAST (Drummond & Rambaut, 2007), using datasets of plastid markers assembled previously, as described in Preece et al. (2015).

Differences in species means were tested using generalized least squares, using the pgls function in the caper package (Orme, 2013). Differences in plant traits between crops and their progenitors were tested as a fixed effect, for example: mod <− pgls(ln.yield~status, data = dat, λ = “ML”). All variables were natural log (ln) transformed, apart from percentage reproductive biomass and harvest index which were left untransformed, and number of tillers per plant which was square‐root transformed. In the results section, we show effect sizes and *p*‐values from the pgls analysis.

For analyses of plant survival and tiller number over time we performed mixed effects models using the lme function in the nlme package, with percentage plant survival logit transformed and tiller number natural log transformed before analysis. To quantify the effect of intraspecific competition on tiller number per plant and harvest index, we compare our data from this study with those from a previous paper of ours (Preece et al., 2015), which utilized the same phylogenetic analysis. Furthermore, we calculated the reduction in harvest index (as (HI as individual – HI in stand)/HI as individual), and used a pgls analysis to see if this differed between crop progenitors and other wild species.

## 3 RESULTS

### 3.1 Yield and harvest index in stands

**Figure 2:**
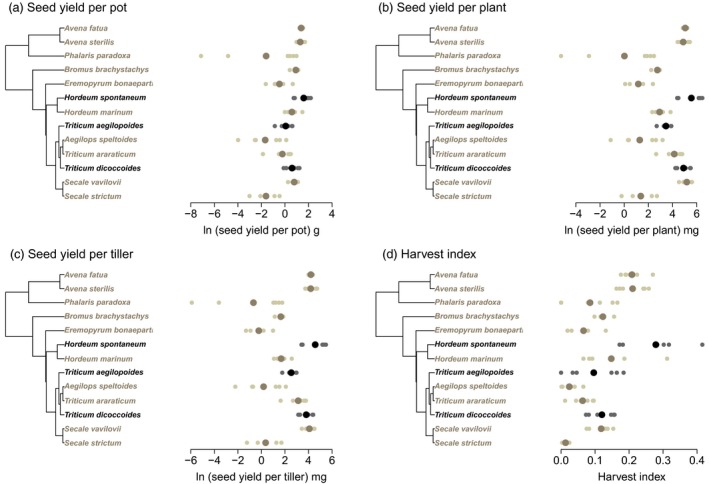
Seed yield in the equal seed mass experiment as natural‐logged values (a) per pot, (b) per plant, (c) per tiller and (d) harvest index shown for crop progenitors and related wild species in relation to the phylogenetic tree. Crop progenitors are shown in black and related wild species in pale brown. Small points indicate individuals and larger points are species means [Colour figure can be viewed at http://wileyonlinelibrary.com]

In the equal seed mass experiment, species average stand yield was 2.3 ×  higher in crop progenitors than other wild species (using species means from the progenitor short list), but this was not a significant difference (Figure 2a and Table 1). Similarly, the seed yields per plant and per tiller were higher for crop progenitors in both cases (by 5.1 and 6.4 ×  respectively), but these differences were also not statistically significant (Figure 2b,c). However, crop progenitors did have a significantly higher HI (seed yield/above‐ground biomass), which was 0.218 in progenitors and 0.124 in other wild species (*p *< .05, *F*_1,10_ = 6.525) (Figure 2d and Table 1). When the less conservative long list of crop progenitors was used, yields at the stand, plant and tiller levels were significantly higher in crop progenitors, as was HI (*p *< .0001 in all cases). Stand yield varied among the species within both groups, ranging between 1.2 and 5.7 g in crop progenitors and 0.3 and 4.0 g in other wild species. There was no difference in height between crop progenitors and other wild species (Table 1).

**Table 1:** Analysis of differences between crop progenitors and related wild species for a range of traits in the equal seed mass experiment. Results are shown for the short and long lists of crop progenitors and show the *p*‐values from the pgls analysis

Trait	Short list of progenitors	Long list of progenitors
ln (seed mass)	NS	**<0.0001**Progenitors larger
ln (biomass)	NS	NS
Percentage rep mass	NS	**<0.0001**Progenitors higher
ln (yield per pot)	NS	**<0.0001**Progenitors higher
ln (yield per plant)	NS	**<0.0001**Progenitors higher
ln (yield per tiller)	NS	**<0.0001**Progenitors higher
HI	**<0.05** Progenitors higher	**<0.0001**Progenitors higher
sqrt (tillers per plant)	NS	NS
ln (seed number per plant)	NS	NS
ln (seed number per tiller)	NS	**<0.05**Progenitors higher
ln (height)	NS	NS
Time to flower	NS	NS

NS = non significant, and numbers in bold indicate a significant difference between crop progenitors and other wild species.

**Figure 3:**
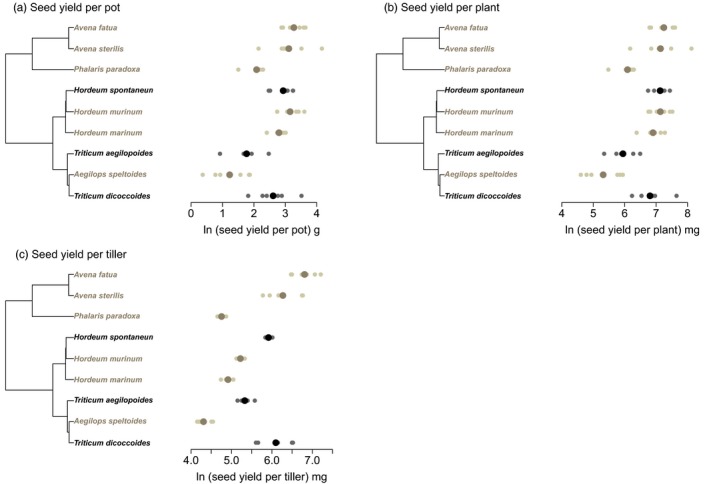
Seed yield in the equal planting density experiment shown (a) per pot, (b) per plant and (c) per tiller, for crop progenitors and related wild species in relation to the phylogenetic tree. Crop progenitors are shown in black and related wild species in pale brown. Small points indicate individuals and larger points are species means [Colour figure can be viewed at http://wileyonlinelibrary.com]

The equal planting density experiment gave similar results, as potential stand yield was not significantly different between crop progenitors and other wild species. In this experiment, tiller mass (seed yield per tiller) was higher in crop progenitors (3.0 × higher, *p *< .05, *F*_1,7_ = 8.97) (Figure 3 and Table 2). Potential yield was higher in this experiment than the actual yield measured in the equal planting mass experiment, either because seed set was overestimated or as a result of the generally lower sowing densities.

**Table 2:** Analysis of differences between crop progenitors and related wild species for a range of traits in the equal planting density experiment

Trait	Short list of progenitors
ln (yield per pot)	NS
ln (yield per plant)	NS
ln (yield per tiller)	**<0.01** Progenitors higher
sqrt (tillers per plant)	NS

Crop progenitors had a higher proportion of fertile tillers than the other wild species (57% compared with 50%), but this was not statistically significant. Maximum height also did not differ significantly between crop progenitors and other wild species. However, mean stem diameter was 1.6 ×  times larger in crop progenitors (*p *< .01, *F*_1,7_ = 23.16). Mean survival of individuals declined for all species over the course of the experiment, but the rate did not differ between crop progenitors and other wild species, although they did have different intercepts (*p *< .01, *t*_7_ = 3.7), such that crop progenitors had lower survival than other wild species (Figure S1). Tiller number increased for all species during the course of the experiment but there was no difference between the two groups of species (Figure S2).

### 3.2 Effect of intraspecific competition

**Figure 4:**
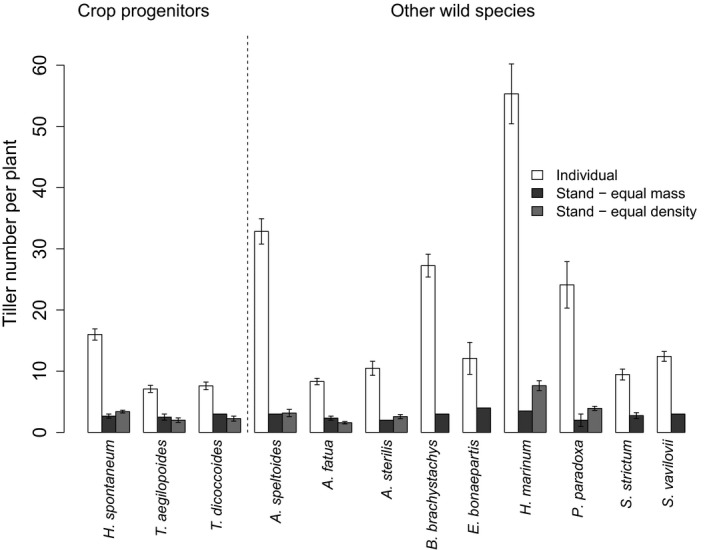
Tiller number for plants growing as individuals (white bars) or in stands derived from an equal seed mass (black bars) or sowing density (grey bars). Error bars (1 *SE*) are shown for all species, except for *Bromus brachytsachys*,* Eremopyrum bonaepartis*,* Hordeum marinum* ssp. *gussoneanum* in the equal mass experiment, which only had one replicate for this measurement. All other instances where there is no visible error bar signify that the error was zero from all replicates having the same value. Note, that *Triticum araraticum* is not included in this graph as it was not included in the experiment with plants grown as individuals

Comparisons of data from the stand experiments with published data from an experiment with plants growing as individuals in the same environmental conditions and same pot size (Preece et al., 2015) enable us to determine the effect of intraspecific competition on harvest traits (summarized in Table 3). When plants were grown individually, the number of tillers per plant was significantly lower in crop progenitors compared with other wild species (Preece et al., 2015), with a mean tiller number of 10.2 for crop progenitors (using the shortlist) and 21.4 for other wild species (and 10.8 and 22.5, respectively, with the progenitor long list). However, tiller number did not differ significantly when plants were grown in stands based on equal seed mass or sowing density. The mean tiller number per plant in the equal seed mass experiment was 2.7 for crop progenitors and 2.8 for other wild species (and 2.8 for both groups when using the progenitor long list). With equal sowing density, the mean tiller number per plant was 2.6 for crop progenitors and 3.9 for other wild species. This indicates a difference in the effect of competition on tillering between these two groups of species, with the advantage that the other wild species have as individuals in terms of tiller number disappearing when in stands (Figure 4).

**Table 3:** Summary of the effects of growing crop progenitors and related wild grasses in stands of equal seed mass or equal seed density, compared with individuals removed from competition

Trait	Grown individually	Stand – equal mass	Stand – equal density
ln (yield per pot)	—	NS	NS
ln (yield per plant)	NS	NS	NS
ln (yield per tiller)	NS	NS	Progenitors larger
Harvest index	NS	Progenitors higher	—
sqrt (tillers per plant)	Progenitors fewer	NS	NS

**Figure 5:**
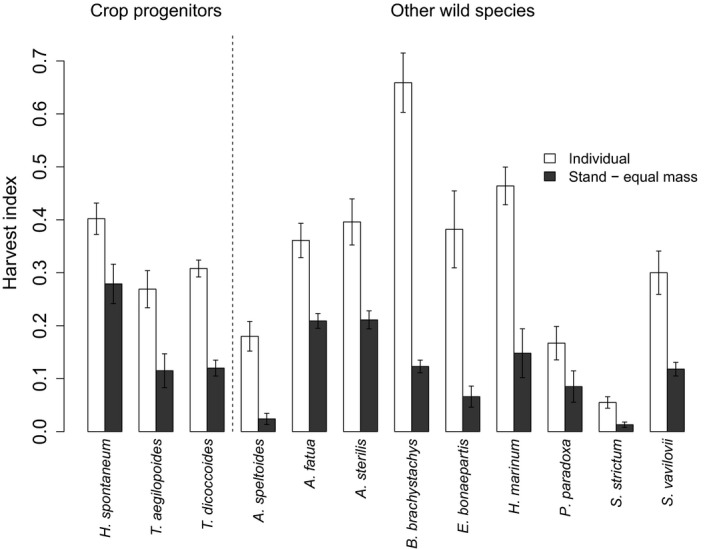
Harvest index for all species in the equal seed mass experiment compared with plants grown as individuals (white bars) or in stands (black bars). Note that final biomass was not measured in the equal planting density experiment, precluding a calculation of HI. Note that *Triticum araraticum* is not included in this graph as it was not included in the experiment with plants grown as individuals

Harvest index (HI) was also affected by intraspecific competition, with HI reduced for all species when growing in stands (Figure 5). There was a significantly greater percentage reduction in HI under competition for other wild species, compared with crop progenitors (*p *< .01, *F*_1,10_ = 13.54), which was 36.2% for crop progenitors and 62.2% for other wild species. When plants were grown as individuals in the previous study (Preece et al., 2015), HI did not differ between crop progenitors and other wild species. Mean HI (calculated from raw data) was 0.326 for crop progenitors and 0.330 for other wild species (and 0.320 and 0.333 respectively using the progenitor long list). However, in stands, as already mentioned, HI was significantly higher in crop progenitors (Table 1), with mean HI (calculated from raw data) being 0.171 for crop progenitors and 0.111 for other wild species (and 0.158 and 0.110 respectively using the long list).

## 4 DISCUSSION

Harvest index (HI) differs between crop progenitors and closely related wild species, when plants grow in stands. Whilst there are signs that yield per unit area may also differ between the two groups of species, HI seems to show larger and more consistent differences. Importantly, this difference in HI is seen only when plants experience intraspecific competition, because competition impacts HI less severely in crop progenitors than other wild species. This disparity, between results from experiments with plants grown as individuals or under competition, shows the importance of experiments that grow plants in a range of different conditions. We also provide evidence that the size of tillers on each plant is a characteristic that separates crop progenitors from closely related wild species.

The significance of these findings for interpreting how and why particular species were domesticated depends on the density at which seed was sown during early attempts at cultivation were conducted. This is obviously unknown but can potentially be inferred from archaeobotanical data. Evidence from the weed floras accompanying the archaeological remains of cereals from later periods, where the archaeobotanical evidence is more plentiful, suggests that in Early Neolithic Europe (some 2,000–3,000 years after the origins of agriculture in southwest Asia) cultivation of cereals seems to have been conducted on a “garden” scale. This was quite unlike that of the modern cereal field, with seed probably sown less densely to facilitate weeding between plants (Bogaard, 2004, 2005; Kreuz & Schäfer, 2011). For these reasons, experiments on individually grown plants may shed more light on the origins of agriculture, than those conducted on plants grown in stands which may be more relevant to the gathering of grain from wild plant stands, or to later developments in agriculture as crop evolution progressed into the late Neolithic and beyond. There is archaeological and genetic evidence to suggest that domestication was an extended process stretching across millennia (Brown, Jones, Powell, & Allaby, 2009; Fuller et al., 2012; Willcox, 2005) and factors relevant for understanding the earliest cultivation might differ from those that promoted the subsequent developments in agriculture. Additionally, the process of crop domestication in southwest Asia may or may not be representative of domestication in other geographic areas.

### 4.1 The importance of high harvest index for crop progenitors

Harvest index (HI) is a way of measuring crop production that has been used by agronomists for decades (Donald, 1962) and is the ratio of the yield of grain to the total plant biomass. High HI is known to be a key trait of modern day crops (Aranjuelo et al., 2013; Araus, Slafer, Reynolds, & Royo, 2002; Hay, 1995; Parry et al., 2010), and we now find that it also appears to be influential in single species stands of crop progenitors. Analysing the data in a phylogenetic context allowed us to account for the relatedness between species and to determine the extent of any differences between the two groups of species. However, care needs to be taken in interpreting these differences in an archaeological context. The findings show that, within particular groups of related species with common characteristics (e.g. *Triticum* and *Aegilops* species, or the genus *Hordeum*), those which were eventually domesticated (the crop progenitors) have greater HI than those which were not. HI does not, however, appear to be the only characteristic distinguishing the species that became domesticated from the wider pool of wild grasses available to early cultivators: although the difference between crop progenitors and other wild species is significantly different when analysed in a phylogenetic context, the HI of the progenitor species encompasses a similar range of values to that of the other wild species. In our attempts to explain the domestication of our earliest cereal crops, therefore, the search for a common suite of characteristics shared by southwest Asian cereal progenitors, indicative of strong selection forces, must extend beyond the confines of yield and harvest characteristics (see, for example, Cunniff et al., 2014; Milla, Osborne, Turcotte, & Violle, 2015; Preece et al., 2015).

Another indication that HI cannot have been the only characteristic determining which species were domesticated is seen in the case of *Avena* (wild oat). The two *Avena* species both have higher HI (and total seed yield) than the three wheat species (two known progenitors and *T. araraticum*, a possible progenitor). This raises the question of why oat was domesticated later than wheat and barley, and suggests either that there were other, more important, factors that determined the domestication process, or that multiple factors, of which HI may be one, provide alternative explanations across different species at different times. Wild oats were stored in large quantities at Gilgal (in the Jordan valley) before agriculture developed in the Fertile Crescent (Weiss, Kislev, & Hartmann, 2006), implying that people gathered this species from extensive wild stands or cultivated wild oats. However, despite this early exploitation, oats did not become domesticated alongside wheat and barley species during the Neolithic in the Fertile Crescent, and were only domesticated later (probably in Europe) (Weiss et al., 2006). A number of factors might explain this difference among species, including competitive ability, nutritional value or agricultural or culinary practices (for example, the lower gluten content of oat rendering it less suitable for bread‐making than wheat or barley (cf. Fuller & Rowlands, 2011; Haaland, 2007). Differences in soils may also play a role, with wheat and barley tending to be grown on relatively fertile soils, while oats (and rye) are normally grown on poorer, more marginal land (Belderok, 2000; Holland, 1997).

Greater HI indicates a higher proportion of edible seed relative to biomass, and can therefore be increased through a larger size or number of seeds or tillers, and/or by decreases in the size or number of stems and leaves. In our studies, greater HI is most related to an increase in reproductive biomass, as we see higher seed mass per tiller in crop progenitors. We note that, in our equal sowing density experiment, stem diameter was larger in crop progenitors, so a decrease in vegetative biomass does not seem to have such an important role. Harvest index has increased during the evolution of crops and the Green Revolution, with modern crops having HI values of around 0.6, which is three and a half times more than that recorded for crop progenitors in this study. In fact, gains in yield over the last few decades are mostly due to increases in HI, but there are indications that the upper limit is being reached (Long, Zhu, Naidu, & Ort, 2006; Richards, 2000). Our data show that this trend for selection on high HI, which persisted through 20th century breeding programs, follows a pattern that was beginning during the process of domestication.

The concept of harvest index was first discussed by Donald (1962) who, in later work, proposed the idea of crop “ideotypes”, which are model plants with ideal characteristics to increase grain production (Donald, 1968). A key part of this work was the suggestion that ideal crops should be weak competitors (relative to their mass) otherwise overall yield would be decreased. Plant ecological knowledge tells us that trade‐offs between different traits are commonplace, and when thinking about agricultural systems, one of the most useful trade‐offs to consider may be that between optimal individual performance and optimal population performance, with high stand yield achieved by low individual fitness (Weiner, 2017). In modern agricultural systems with adequate water and nutrients, the most limiting resource is light, so for an individual plant to be successful it should grow tall and produce many leaves. However, this reduces the overall yield of the crop in the so‐called “tragedy of the commons” which can be described using evolutionary game theory (Anten & Vermeulen, 2016; Zhang, Sun, & Jiang, 1999), whereby individual plants gain from this behaviour, but the costs are shared among the whole population (in this case, the crop stand). Thus Donald's (1968) proposal for an ideal wheat plant would be that it is short in stature, with a low number of leaves and a large ear (Donald, 1968), as in modern high‐yielding varieties, which have a high HI.

### 4.2 Tiller size and total seed yield

The data from the equal seed mass experiment indicated that the total seed mass per tiller was nearly twice as high in crop progenitors as in closely related wild species, and this matches previous findings for plants grown individually (Preece et al., 2015). In terms of early seed gathering, even if total seed yields were similar between two species, if one packaged these into a smaller number of large ears, this could have been a more easily harvested and processed food source, leading to selection for larger tillers if collected grain was re‐planted elsewhere. During the domestication process, selection has tended to enlarge inflorescences in cereals (Harlan, de Wet, & Price, 1973). Our data suggest that this characteristic might also have been an important factor in narrowing the range of species exploited during the transition to agriculture.

Total seed yield does not seem to be the main determining factor for the selection of crop progenitors, given our previous data on individuals (Preece et al., 2015), and now in stands. However, this trait cannot be completely discounted because, in this current study, when the long list of potential crop progenitors is used, they do have significantly higher yield both at the level of the stand and the plant. Additionally, higher yield in crop progenitors has previously been estimated among a smaller range of wild species (Cunniff et al., 2014). It may be that the yield advantage of crop progenitors is not always apparent, and is contingent on the particular set of species used for comparisons, or requires particular conditions that are yet to be identified. For example, up until now, experimental studies have only followed one year of growth, which reduces the possibility of beneficial effects on crop progenitors from the characteristics relating to the soil. Plant roots produce exudates that can have a wide range of effects (Bais, Weir, Perry, Gilroy, & Vivanco, 2006), including allelopathy (chemical inhibition of one plant by another), which can disrupt germination or growth of competitors (Bertin, Yang, & Weston, 2003; Kong, Li, Hu, Xu, & Wang, 2006). Alternatively, exudates can increase the success of the species, through induced herbivore resistance (Glinwood et al., 2003) or changes in soil nutrient availability, such as to increase phosphate and micronutrient availability (Bais et al., 2006). Thus, yield differences between crop progenitors and other wild species may only be apparent after a number of consecutive years in the same soil. Nonetheless, yield differences between crop progenitors and other wild species are less consistent than other harvest characteristics.

### 4.3 Changes in tillering under competition

Through comparisons with previous work where plants were grown in isolation, we observed that crop progenitors and other wild species differed in how tiller number is affected by competition. When grown individually, other wild species are able to produce a much higher number of tillers than crop progenitors, and therefore produce a larger quantity of seed when conditions are favourable. However, in a single‐species stand, individuals within both groups of species produce an average of three tillers per plant, such that the advantage of the other wild species disappears. This offers support to the hypothesis that tillering of progenitors and their cultivated descendants would be less affected by intraspecific competition than tillering of other wild species.

As cultivation progressed and people became more dependent on domesticated species, sowing density may have increased and, if so, plants would not have been growing in anything close to “ideal” conditions with low competition. Therefore, in later agricultural periods, more importance should be given to the tiller data from the experiments under competition, which indicate that tiller size is more important than tiller number as a distinguishing characteristic between these two groups of species. Data for tiller number during the course of the equal planting density experiment did not differ between crop progenitors and related wild species during stand development. However, there were two distinct groups of species: one group with generally lower and more stable tiller numbers containing the three crop progenitors plus the two *Avena* species, and a second group of the remaining wild species which shows tiller number increasing throughout the experiment. Both of the *Avena* species included in the experiments are thought to have contributed to the gene pool of domesticated oat (which was domesticated significantly later than wheat and barley; Zohary et al., 2012), so we may speculate that consistent tiller number is characteristic of cereal crops, but is not a defining characteristic of early crop progenitors. Research into grass architecture using phylogenetic and genomic methods has shown similarities between different cereal crops, with the tendency for crops to have taller, straighter growth and apical dominance, in contrast to their wild relatives (Doust, 2007; Fuller et al., 2010). Improvements to tiller economy are currently underway in modern crop breeding, with particular interest in the *tin* gene in wheat, which when manipulated, produces plants with fewer tillers, larger spikes and more seeds than wildtype in drought and elevated CO_2_ conditions (Dias de Oliveira, Siddique, Bramley, Stefanova, & Palta, 2015; Mitchell, Rebetzke, Chapman, & Fukai, 2013; Tausz‐Posch et al., 2015).

## 5 CONCLUSIONS

Our results show that cereal crop progenitors produce a greater proportion of harvestable material in stands than closely related wild grass species but the same number of tillers, indicating that they are less affected by intraspecific competition than close relatives. These data imply a difference in the harvest characteristics in wild stands of cereal progenitors to those of closely related wild grain species. Though this may have played some part in determining which species were subsequently used during early experiments with cultivation, there is large variation in harvest index among species, with some wild species that were not domesticated having high allocation to grains. This suggests that factors other than HI may have had a greater effect on which species were selected during the process of domestication, or that HI was one of multiple factors contributing to the selection of crops from the pool of wild grain species.

## AUTHORS' CONTRIBUTIONS

C.P., C.P.O., M.R., G.J. and M.C. conceived the ideas and designed methodology; C.P., N.C. and G.W. collected the data; C.P. and N.C. analysed the data; C.P., N.C., G.J. and C.P.O. led the writing of the manuscript. All authors contributed critically to the drafts and gave final approval for publication.

## DATA ACCESSIBILITY

Seed accession data uploaded as online supporting information.

Phylogenetic data and species trait data uploaded to figshare: https://doi.org/10.6084/m9.figshare.5526997.v3 (Preece, 2017).
