# Supplementary material for: Cereal progenitors differ in stand harvest characteristics from related wild grasses
Source: J Ecol. 2017 Nov 27;106(3):1286–97. doi: 10.1111/1365-2745.12905 (PMC5947309; doi:10.1111/1365-2745.12905)

Supporting Information for:

**Cereal progenitors differ in stand harvest characteristics from related wild grasses**

Catherine Preece, Natalie F Clamp, Gemma Warham, Michael Charles, Mark Rees, Glynis Jones and Colin P Osborne.

Table S1. List of grass accessions used in the equal seed mass experiment, including the seed bank it was obtained from, country of origin and mean initial seed mass in mg. Seed banks used were the USDA Germplasm Resources Information System (GRIN) and IPK Gatersleben Genebank (IPK).

| Species | Group | Seed bank | Accession  numbers | Country  of origin | Mean seed mass (mg) |
| --- | --- | --- | --- | --- | --- |
| *Aegilops speltoides* Tausch | Other wild | GRIN | PI 170204  PI 487231 | Turkey  Syria | 7.03  8.47 |
| *Avena fatua* L. | Other wild | GRIN | PI 126259  PI 173584 | Afghanistan  Turkey | 16.66  14.72 |
| *Avena sterilis* L. | Other wild | GRIN | PI 326955  PI 134251 | Israel  Afghanistan | 16.92  12.33 |
| *Bromus brachystachys* Hornung | Other wild | GRIN | PI 220582 | Afghanistan | 2.47 |
| *Eremopyrum bonaepartis* (Spreng.) Nevski | Other wild | GRIN | PI 227343  PI 227345 | Iran  Iran | 1.50  2.21 |
| *Hordeum marinum* subsp. *gussoneanum* (Parl.) Thell. | Other wild | GRIN | PI 204582  PI 220522 | Turkey  Afghanistan | 4.13  4.15 |
| *Hordeum vulgare* L. subsp. *spontaneum* (K. Koch) Thell. | Progenitor | GRIN | PI 282628  PI 466114 | Israel  Syria | 15.61  27.77 |
| *Phalaris paradoxa* L. | Other wild | GRIN | PI 202684  PI 380964 | Turkey  Iran | 2.69  1.74 |
| *Secale strictum* (C. Presl) C. Presl | Other wild | GRIN | PI 383756  PI 240286 | Turkey  Turkey | 6.60  9.43 |
| *Secale vavilovii* Grossh. | Putative progenitor | GRIN | PI 284842  PI 573649 | Hungary  Afghanistan | 36.24  29.41 |
| *Triticum araraticum* Jakubz. | Putative progenitor | IPK | TRI 18513  TRI 17220 | Iraq  Iraq | 36.36  26.67 |
| *Triticum monococcum* L. subsp. *aegilopoides* (Link) Thell. | Confirmed progenitor | GRIN | PI 427452  PI 245726 | Turkey  Turkey | 14.02  10.64 |
| *Triticum turgidum* L. subsp. *dicoccoides* (Körn. ex Asch. & Graebn.)Thell. | Confirmed progenitor | GRIN | PI 300989  PI 428022 | Israel  Turkey | 31.35  28.39 |

Table S2. List of grass accessions used in the equal seed density experiment, including the seed bank it was obtained from, country of origin and mean initial seed mass in mg. Seed banks used were the USDA Germplasm Resources Information System (GRIN).

| Species | Group | Seed bank | Accession  numbers | Country  of origin | Mean seed mass (mg) |
| --- | --- | --- | --- | --- | --- |
| *Aegilops speltoides* Tausch | Other wild | GRIN | PI 170204 | Turkey | 6.75 |
| *Avena fatua* L. | Other wild | GRIN | PI 544659  PI 173584 | USA  Turkey | 15.72  12.26 |
| *Avena sterilis* L. | Other wild | GRIN | PI 309527  PI 412641  PI 309424  PI 311656  PI 326955 | Israel  Turkey  Israel  Israel  Israel | 14.29  15.42  12.84  17.66  18.74 |
| *Hordeum marinum* subsp. *gussoneanum* (Parl.) Thell. | Other wild | GRIN | PI 220522  PI 204582 | Afghanistan  Turkey | 3.69  3.64 |
| *Hordeum murinum subsp. glaucum* (Steud.) Tzvelev | Other wild | GRIN | PI 204874  PI 244762 | Turkey  Iran | 2.90  4.08 |
| *Hordeum vulgare* L. subsp. *spontaneum* (K. Koch) Thell. | Confirmed progenitor | GRIN | PI 282628  PI 466114  PI 554424  PI 391107  PI 596285  PI 420910  PI 236388 | Israel  Syria  Turkey  Israel  Turkey  Jordan  Syria | 14.24  26.70  45.10  30.50  30.75  20.45  28.40 |
| *Phalaris paradoxa* L. | Other wild | GRIN | PI 170618 | Turkey | 1.28 |
| *Triticum monococcum* L. subsp. *aegilopoides* (Link) Thell. | Confirmed progenitor | GRIN | PI 245726  PI 427452  PI 654316  PI 427471  PI 427642  PI 427448  PI 427456  PI 427458 | Turkey  Turkey  Turkey  Turkey  Iraq  Turkey  Turkey  Turkey | 16.60  12.60  10.65  5.00  15.58  10.43  6.20  14.74 |
| *Triticum turgidum* L. subsp. *dicoccoides* (Körn. ex Asch. & Graebn.)Thell. | Confirmed progenitor | GRIN | PI 428022  PI 300989  PI 355455  PI 503315  PI 316905 | Turkey  Israel  Lebanon  Israel  Israel | 28.17  30.99  26.20  31.37  39.99 |

| Species | Yield per pot (g) | Yield per plant (mg) | Yield per tiller (mg) | Harvest Index |
| --- | --- | --- | --- | --- |
| *A.fatua* | 3.97 | 155.88 | 66.81 | 0.209 |
| *A. speltoides* | 0.40 | 7.77 | 2.59 | 0.024 |
| *A. sterilis* | 3.78 | 135.05 | 67.52 | 0.211 |
| *B. brachystachys* | 2.62 | 16.14 | 5.38 | 0.123 |
| *E. bonaepartis* | 0.82 | 4.15 | 1.04 | 0.066 |
| *H.marinum* | 2.10 | 21.72 | 6.21 | 0.148 |
| *H.spontaneum* | 5.68 | 283.93 | 106.47 | 0.279 |
| *P.paradoxa* | 1.25 | 6.19 | 3.10 | 0.085 |
| *S. strictum* | 0.30 | 5.89 | 2.14 | 0.013 |
| *S. vavilovii* | 2.29 | 183.44 | 61.15 | 0.118 |
| *T. araraticum* | 1.02 | 78.45 | 28.53 | 0.063 |
| *T.aegilopoides* | 1.24 | 36.92 | 14.77 | 0.115 |
| *T.dicoccoides* | 2.04 | 151.38 | 50.46 | 0.120 |

Table S3. Mean values of yield per pot (g), yield per plant (mg), yield per tiller (mg) and harvest index (proportion of seed relative to aboveground biomass) for species in the equal seed mass experiment.

Table S4. Mean values of yield per pot (g), yield per plant (mg), yield per tiller (mg) and harvest index (proportion of seed relative to aboveground biomass) for species in the equal sowing density experiment.

| Species | Yield per pot (g) | Yield per plant (mg) | Yield per tiller (mg) |
| --- | --- | --- | --- |
| *A.fatua* | 27.58 | 1480.0 | 940.0 |
| *A. speltoides* | 3.97 | 235.8 | 75.3 |
| *A. sterilis* | 27.26 | 1504.2 | 568.1 |
| *H. marinum* | 16.70 | 1029.1 | 136.8 |
| *H.murinum* | 24.67 | 1309.2 | 185.7 |
| *H.spontaneum* | 19.66 | 1278.3 | 372.2 |
| *P.paradoxa* | 8.30 | 455.0 | 116.6 |
| *T.aegilopoides* | 6.45 | 406.7 | 208.3 |
| *T.dicoccoides* | 15.73 | 1008.3 | 473.2 |

Figure S1. Mean percentage survival for each species throughout the equal stand density experiment. Fitted lines show the survival over time for crop progenitors (solid) and other wild species (dotted). *Hordeum spontaneum* plants were destroyed before the end of the experimental period due to a suspected mildew infection.


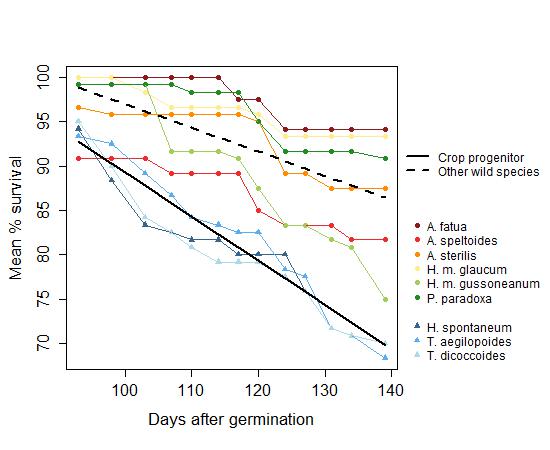


Fig. S2. The change in the number of tillers per plant over time. The fitted line shows the average number of tillers for all species together, as there was no statistical difference between crop progenitors and other wild species. *Hordeum spontaneum* plants were destroyed before the end of the experimental period due to a suspected mildew infection.


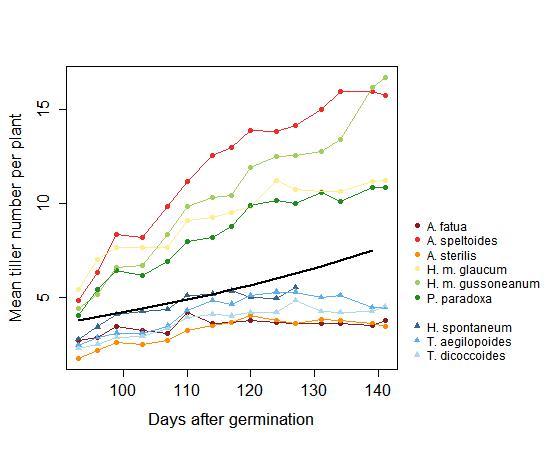

Supplement: Supplementary file 1 [file JEC-106-1286-s001.docx]
